# Development and Validation of a Scoring System for Assessment of Clinical Failure after Pediatric Robot-Assisted Laparoscopic Extravesical Ureteral Reimplantation: A Multi-Center Study

**DOI:** 10.3390/jcm11051327

**Published:** 2022-02-28

**Authors:** Chester J. Koh, Kun Suk Kim, Jonathan A. Gerber, Vinaya Bhatia, Huirong Zhu, Minki Baek, Sang Hoon Song

**Affiliations:** 1 Division of Pediatric Urology, Department of Surgery, Texas Children’s Hospital, Houston, TX 77030, USA; ckoh@bcm.edu (C.J.K.); jonathan.a.gerber@gmail.com (J.A.G.); vvasude@gmail.com (V.B.); 2 Scott Department of Urology, Baylor College of Medicine, Houston, TX 77030, USA; 3 Department of Urology, Asan Medical Center, University of Ulsan College of Medicine, Seoul 05535, Korea; kskim2@amc.seoul.kr; 4 Outcomes and Impact Service, Department of Surgery, Texas Children’s Hospital, Houston, TX 77030, USA; hxzhu@texaschildrens.org; 5 Department of Urology, Samsung Medical Center, Sungkyunkwan University of Medicine, Seoul 06351, Korea; drminkibaek@gmail.com

**Keywords:** vesicoureteral reflux, ureteral reimplantation, robotic surgery, pediatric

## Abstract

We aimed to develop and validate a scoring system as an objective assessment tool for predicting clinical failure after pediatric robotic extravesical ureteral reimplantation. Data for this multi-institutional retrospective cohort was obtained from two tertiary referral hospitals. We defined clinical failure as incomplete radiographic resolution or post-operative febrile UTI. Patients were stratified into low, intermediate, and high-risk groups according to the score. External validation was performed using the model projected to the external validation cohort. An amount of 115 renal units in the development cohort and 46 renal units in the validation cohort were analyzed. The prediction score was calculated with weighted points to each variable according to their regression coefficient as age (year) + BMI + BBD times 10 + VUR grade times 7 + console time (h) + hospital stay times 6. The C-index of our scoring system was 0.850 and 0.770 in the development and validation cohorts, respectively. Clinical failure was significantly different among risk groups: 0% (low-risk), 3.3% (intermediate-risk), and 22.2% (high-risk) (*p* = 0.004) in the development cohort. A novel scoring system using multiple pre- and intra-operative variables provides a prediction of children at risk of failure after robotic extravesical ureteral reimplantation.

## 1. Introduction

Vesicoureteral reflux (VUR) is one of the most common urological conditions diagnosed in the pediatric population [1]. Surgical treatment is indicated in patients with persistent VUR and those at higher risk for febrile urinary tract infection (UTI) or renal scarring [2,3,4]. The goal of extravesical ureteral reimplantation is to achieve VUR resolution by elongating the submucosal ureteral tunnel length with reduced perioperative morbidity, such as bladder spasms and hematuria when compared to the transvesical approach [5]. Robot-assisted laparoscopic extravesical ureteral reimplantation (RALUR-EV) has been reported as one of several options for surgical management of VUR in children [6]. Radiologic resolution rates of VUR and complication rates after RALUR have been reported as comparable to open ureteral reimplantation in previous multicenter studies and reviews [6,7].

Robotic surgery enables more uncomplicated dissection and intracorporeal suturing as well, compared to conventional laparoscopy [8,9,10].

However, there have been reports of suboptimal results with lower success rates and higher complication rates at some centers, which may reflect technical or learning curve differences. There are well-known critical points that need exceptional attention to minimize complications with the extravesical ureteral reimplantation technique, such as a “no-touch” technique and the avoidance of electrocautery during the ureteral dissection. Complications, such as ureteral obstruction, ureteral injury and urine leak have been reported at some centers, similar to those previously reported in open extravesical series [11,12]. We hypothesized that identifying predictive factors for successful VUR resolution after RALUR-EV is needed and will help shorten the learning curve for surgeons and improve outcomes for patients when performed by surgeons at any level of experience. Therefore, we aimed to develop and validate a scoring system as an objective assessment tool for predicting success or failure after RALUR-EV.

## 2. Materials and Methods

### 2.1. Study Population

Data for this multi-institutional retrospective cohort study of RALUR-EV patients was obtained from two tertiary referral hospitals (hospitals A and B). Institutional review board approval was obtained for this retrospective study by the Institutional Review Board of Baylor College of Medicine (Protocol H-33575). This study was performed in accordance with the ethical standards of the Declaration of Helsinki and its later amendments. Because it was a retrospective study, the informed consent was waived by the Institutional Review Board of Baylor College of Medicine. The medical records were reviewed after institutional review board approval. The larger cohort between the two institutions was designated as a development cohort and the other as an external validation cohort. The scoring system and risk model were developed using data from the development cohort. The performance of the risk model was validated using the validation cohort. The indications for RALUR-EV were persistent primary grade II to V VUR, breakthrough urinary tract infections, and/or progression of renal scarring despite the use of antibiotic prophylaxis. We excluded children undergoing a re-do RALUR-EV for recurrent VUR and children with other associated urinary pathology, such as megaureter, ectopic ureter, ureterovesical junction obstruction and periureteral diverticulum. In addition, patients lost to follow-up were excluded from the study.

### 2.2. Surgical Technique and Post-Operative Care

The RALUR-EV technique in this study was previously described by Silay et al. [13]. In brief, da Vinci Si or Xi Surgical System (Intuitive Surgical, Sunnyvale, CA, USA) robotic instruments were used to perform a modified Lich–Gregoir extravesical reimplantation technique. After mobilizing the ureters, detrusor muscle troughs were formed by splitting the detrusor muscle along its new muscle tunnel to create an approximate 5:1 tunnel length to the ureteral diameter ratio. Detrusorrhaphy was performed over the ureter in a top-down or bottom-up manner. A urethral catheter was routinely placed for 1 or 2 days post-operatively. Ketorolac or opioids were administered if necessary. Renal ultrasound was performed at the 1-month mark after surgery, and then at least every 6 months. Voiding cystourethrogram (VCUG) or radionuclide cystogram (RNC) was performed at the 3 to 4-month post-operative follow-up.

### 2.3. Development of Prediction Score and Statistical Analysis

The statistical methods were in accordance with the Transparent Reporting of a Multivariable Prediction Model for Individual Prognosis or Diagnosis statement [14] and the statistical methods for prediction models [15]. We aimed to develop a risk scoring system for the prediction of clinical success after RALUR-EV. In this study, clinical failure was defined as an incomplete radiographic resolution of VUR on follow-up voiding cystourethrogram (VCUG) or radionuclide cystogram (RNC), or post-operative febrile UTI or additional intervention or surgery when follow-up VCUG or RNC was not available. Univariate and multivariate logistic regression analyses were performed to evaluate the risk of persistent VUR after RALUR-EV. We utilized the variables including age, sex, body mass index (BMI), bladder and bowel dysfunction (BBD) status, VUR grade, console time, hospital stay and the number of detrusorrhaphy stitches as the potential predictor candidates of VUR resolution in univariate analysis. Variable selection for the multivariate model was achieved by backward elimination in binary logistic regression analysis. A simplified scoring system was developed following the method of Sullivan et al [16]. Risk factors in the final model were assigned weighted points that were proportional to their β regression coefficient values, and the risk scores were calculated for each patient. Then patients were stratified into low, intermediate, and high-risk categories, which were significantly different in their predictive risk for failure after RALUR-EV. To support the generalizability of our model, we performed an external validation with data from institution B. This was achieved by evaluating the model performance with C-index. All statistical analyses were performed using SPSS version 25 (IBM Corp, Armonk, NY, USA). A two-sided *p*-value < 0.05 was considered statistically significant.

## 3. Results

A total of 77 patients with 115 renal units in the development cohort and 28 patients with 46 renal units in the validation cohort were included in this study (Table 1). In the development cohort, 57 patients (74%) were female, and 38 (49.4%) patients were bilateral cases. The VUR grade was Ⅰ in 12 (10.4%), grade Ⅱ in 16 (13.9%), grade Ⅲ in 51 (44.3%), grade Ⅳ in 29 (25.2%), and grade Ⅴ in 7 (6.1%) renal units. Renal units with VUR grade Ⅰ underwent reimplantation only in bilateral cases with contralateral VUR grade Ⅱ or more. The clinical success rate after RALUR-EV was 93.0%. Patients’ characteristics in the validation group were similar to the development group in age, gender, laterality distribution, total operative time and clinical success rate. However, the validation group showed statistically higher VUR grade, length of stay, follow-up period, and shorter console time with similar success rates (Table 1).

### 3.1. Prediction Model and Scoring System Development with Regression Analysis

We used univariate and multivariate binary logistic regression analyses in the development cohort to determine the potential variables to predict surgical failure after RALUR-EV (Table 2 and Table 3). Age at surgery, BMI, VUR grade, console time and hospital stay were included in our model to predict surgical failure. These variables were assigned to weighted points according to their β (regression coefficient) values based on the logistic regression analysis. The simple scoring system was calculated as follows: age (year) + BMI + BBD times 10 + VUR grade times 7 + console time (h) + hospital stay times 6. The Hosmer–Lemeshow goodness-of-fit test indicated that the prediction model and scoring system were well-calibrated (*p* = 0.976). Patients were stratified into low-risk (<52 points), intermediate-risk (52–70 points), and high-risk groups (≥71 points), with a risk of failure after surgery at the time of the first VCUG or RNC follow-up for each group.

### 3.2. Risk Model Performance Validation

**Figure 1:**
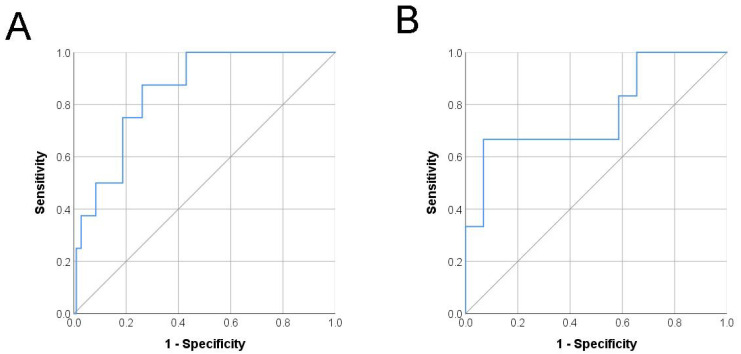
Receiver operating curve analysis of the VUR resolution prediction scoring system for the development cohort (**A**), area under curve = 0.850, 95% CI = 0.744–0.957, *p* = 0.001) and the validation cohort (**B**), area under curve = 0.770, 95% CI = 0.534–1.000, *p* = 0.040).

Risk group stratification showed that the low-risk group had a 100% resolution in the development and validation group. However, patients in the high-risk group showed a poor resolution rate (Table 4). The ROC curve also showed a good discrimination potential of the scoring system both in the development and validation cohort (Figure 1). In the development cohort, the C-index of the simple scoring system was 0.850, with a 95% CI, 0.744–0.957 (*p* = 0.001). The sensitivity and specificity were 75.0% and 81.3%, using the cutoff value of 71.9 points. In the validation cohort, the C-index of the scoring system was 0.770, with a 95% CI, 0.534–1.000 (*p* = 0.040). The sensitivity and specificity were 66.7% and 93.1%, using the cutoff value of 83.0 points.

### 3.3. Risk Prediction with Only Pre-Operative Variables

**Figure 2:**
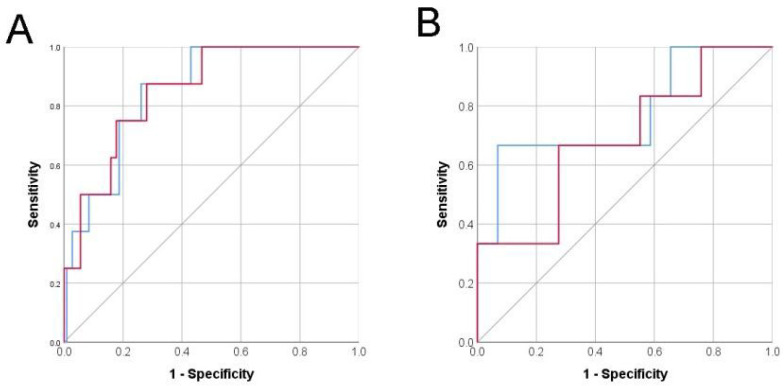
Receiver operating curve analysis with (**A**), blue line, area under curve = 0.850, 95% CI = 0.744–0.957, *p* < 0.001, (**B**), blue line, area under curve = 0.770, 95% CI = 0.0534–1.000, *p* = 0.040) or without (**A**), red line, area under curve = 0.850, 95% CI = 0.737–0.964, *p* < 0.001, (**B**), red line, 0.690, 95% CI = 0.448–0.931, *p* = 0.149) intra- and post-operative variables in the development (**A**) and validation (**B**) cohorts.

When we selected only pre-operative variables for the prediction model, such as age, BMI and VUR grade at the time of surgery and assigned weighted points according to their β coefficient, the score was calculated as age (year) + BMI + BBD times 6 + VUR grade times 5. This new model showed a similar discriminatory ability of the scoring system in the development cohort (Figure 2A) with a category-based net reclassification improvement (NRI) of 0.495, with 0% and 49.5% of patients who failed and succeeded correctly reclassified by the model with only pre-operative variables (Supplementary Table S1). However, in the validation cohort, the discriminatory ability was less significant, with a C-index of 0.690 (95% CI 0.448–0.931, *p* = 0.149) (Figure 2B).

## 4. Discussion

In this study, we developed and validated a simple scoring system predicting surgical failure after RALUR-EV. This scoring system showed an excellent predictive ability with the C-index of 0.850 in the development cohort and 0.770 in the validation cohort. It is noteworthy that even though the two cohorts had different clinical characteristics in terms of pre- and intra-operative variables, the scoring system demonstrated high performance in both groups. Stratifying this score into three distinct risk categories, surgeons will be able to quickly identify patients who are at risk of failure after RALUR-EV. The simplified scoring method used in this study is one of the useful methods to present the expected probability of an outcome, which can be conveniently applied to clinical practice [15,16]. This approach has been used widely in the field of medicine and validated for its efficacy in urological, cardiovascular, and oncological diseases [17,18,19]. To the best of our knowledge, this is the first scoring system to predict failure after surgical treatment of VUR.

Our scoring system included age, BMI, BBD, VUR grade, console time and hospital stay based on the multivariate analysis to determine risk factors for failure. The majority of these variables had been reported to be risk factors for predicting surgical failure in previous studies. Herz et al. showed that age less than 3 years, VUR grade more than 3, pre-operative BBD were significant risk factors to predict surgical failure after bilateral RALUR-EV [20]. In a multi-institutional study, longer operative times and length of stay were associated with radiographic failure on univariate analysis, but after controlling for age, VUR grade and operative time were the only significant factors associated with radiographic failure [21]. On the contrary, Akhavan et al. reported that failure after RALUR-EV was not associated with older age, pre-operative VUR grade, bilaterality or voiding dysfunction [22]. In a prospective multicenter study on RALUR-EV by Boysen et al., no patient or technical factors were found to be associated with radiographic failure [6].

Comparing prediction models with or without intra- and post-operative variables, we found that the discriminative power was higher when we include intra- and post-operative variables in the prediction model (Figure 2). Indeed, outcomes of RALUR-EV were reported to be dependent on surgical technique and a surgeon’s experience level [23]. Gundeti et al. demonstrated that intra-operative technical factors, such as ureteral advancement; a 4–5 cm long detrusortomy, regardless of pre-operative VUR grade; and ureteral stay stitch increased surgical success rates in their cohort [24]. Even experienced laparoscopic surgeons reported the inevitable learning curve associated with RALUR-EV and noted improved outcomes after at least the first five to seven cases [25]. Given that all the surgeries in the development cohort were performed by a single surgeon who had passed the learning curve with greater than 50 robotic cases before this study, console time in the prediction model may reflect the surgical difficulty of each case. Therefore, by including console time in the prediction model, we can usefully evaluate the surgeon’s experience level factor with our model, just as with any other group having a diversity of technical characteristics.

BBD is a well-known risk factor for febrile UTI in children [26], but has also been associated with suboptimal surgical outcomes [27]. After RALUR-EV, BBD has been described as a significant risk factor for failure in bilateral cases [20]. Therefore, we included BBD in the final regression model for the scoring system, although it was not a statistically significant variable in the univariate analysis. The BBD incidence in the development cohort in this study was 46.1%, which was similar to the BBD incidence of 45.5% in the multi-institutional study [6]. However, the BBD incidence in the validation cohort was significantly lower at 10.9%. This difference may be secondary to the lack of standardization of BBD diagnostic criteria and treatment protocols before surgical correction of VUR between the two cohorts in this study. For the diagnosis of BBD, the two hospitals used similar questionnaires to diagnose BBD, such as Vancouver Symptom Score for Dysfunctional Elimination Syndrome (VSSDDES), DVSS, a modified version of DVSS, and the Bristol Stool Scale [28,29,30,31]. Therefore, we hypothesize that the difference in BBD rates from the two centers at the time of surgery resulted from the different treatment protocols for BBD at the time of surgical correction. However, the heterogeneity of our study population appears to strengthen the generalizability of our scoring system.

Validation is a critical step in generating a new predictive model. Although we reviewed several clinical guidelines from multiple urological associations for the treatment of VUR of pediatric patients [2,3], every pediatrician and pediatric urologist may develop variations in their treatment algorithms to decide when and how to treat a patient with VUR based on available evidence and patient/parent preferences. However, generalizability is often needed to develop a prediction model for the treatment of VUR. Therefore, we utilized external validation, rather than internal validation, in an independent group of patients. In previous studies, it was suggested that a minimum of 100 events is required for external validation of a prediction model [32]. However, it is highly unlikely that one will be able to obtain a cohort of RALUR-EV with more than 100 failures due to the high success rates of the procedure and relatively low case volumes when compared to other robotic procedures, such as prostatectomy. Although we demonstrated a proper validation of our model in a relatively small number of patients, further external validation may be necessary to obtain improved reliability and accuracy.

RALUR-EV has been reported as an effective surgical option for primary VUR in pediatric patients [7]. The success rate in our study was comparable to those of previous studies for RALUR-EV [6,22,24] as well as for open reimplantation [33]. Thus, our study provides additional evidence to the literature that RALUR-EV can be a useful and minimally invasive option for the treatment of VUR in pediatric patients. The success rate in the validation group was slightly lower than in the development group, although not statistically significant (Table 1). This success rate of 87.0% was comparable to that of which from a developing stage of expertise in another cohort [24]. This suggests that our prediction model may be most useful for surgeons who are in the learning curve stage of their RALUR-EV experience.

This study has limitations that are notable. First, selection bias may be possible due to small study population numbers. Even though some patients have two renal units, the total number could still be small for building a prediction model. Enrollment of additional patients and reaffirmation of the power of the regression model is warranted. Second, we selected variables into the multiple regression model based on the univariate analysis significance. Although univariate prefiltering is commonly used in medical science, this method might have wrongly excluded potentially essential variables [34]. Third, a radiographic study to confirm the resolution of VUR in all patients was not performed, in part due to the evolution of our management protocol, where a post-operative VCUG or RNC is no longer routinely obtained, similar to the open reimplantation experience. While the routine objective assessment of radiographic resolution after RALUR-EV has confirmed the success in previous reports and in our early experiences, a VCUG or RNC was only offered to the initial chronological subset (approximately ½) of the patients, or when suspicion of a febrile UTI was noted for patients in the development cohort [13,25]. As VCUG or RNC is currently avoided in most pediatric patients undergoing open ureteral reimplantation [35], we used the assumption that any study investigating the efficacy and validity of RALUR-EV should use clinical success or failure if a routine post-operative VCUG / RNC is not obtained.

In summary, while limitations to our study include its retrospective design and relatively small patient numbers for the development of a scoring system, we have shown that the scoring system was validated in a heterogeneous group, and therefore can be applied to various populations with diverse characteristics. We suggest that more attentive and strict follow-up of those patients at a high risk of clinical failure may be warranted to maximize the surgical outcome.

## 5. Conclusions

A novel VUR resolution scoring system using multiple pre- and intra-operative variables including patient’s age, BMI, BBD, VUR grade, console time and hospital stay provides a prediction of children at risk for failure of VUR resolution after RALUR-EV.

## Figures and Tables

**Table 1:** Patient demographics and pre-operative data.

Parameters	Development Cohort	Validation Cohort	*p*-Value
No. Patients	77	28	
Total Ureter Units	115	46	
Gender (%)			
Male	20 (26.0)	11 (39.3)	0.122
Female	57 (74.0)	17 (60.7)	
Median age in years (range)	5.5 (1–16)	6.5 (0.3–46)	0.179
Laterality (%)			
Left	20 (26.0)	5 (17.9)	0.054
Right	19 (24.7)	5 (17.9)	
Bilateral	38 (49.4)	18 (64.3)	
BBD	53 (46.1%)	5 (10.9%)	<0.001
VUR Grade (%)			
I	12 (10.4)	1 (2.2)	0.048
II	16 (13.9)	8 (17.4)	
III	51 (44.3)	13 (28.3)	
IV	29 (25.2)	18 (39.1)	
V	7 (6.1)	6 (13.0)	
Median total operative time (min)	196 (98–273)	195 (120–360)	0.927
Median console time (min)	148 (75–240)	105 (75–225)	<0.001
Median length of stay in days (range)	1.0 (1–6)	2 (2–7)	<0.001
Median follow-up in months (range)	4.3 (1–19)	10 (1–41)	<0.001
Follow-up VCUG ^1^ or RNC ^2^ conducted (%)	66 (57.4)	37 (80.4)	<0.001
Clinical success (%)	107 (93.0)	40 (87.0)	0.227

^1^ Voiding cystourethrography; ^2^ radionuclide cystogram.

**Table 2:** Univariate analysis according to clinical success or failure of renal units in the development cohort.

Variable	Development Cohort		
	Success (*n* = 107)	Failure (*n* = 8)	*p*-Value
Age	4.9 (1–16.2)	8.75 (6–13)	0.007
Gender			1.000
Female	81 (75.7%)	6 (75.0%)	
Male	26 (24.3%)	2 (25.0%)	
BMI ^1^	17.5 (13.4–41.4)	20.5 (15.2–24.3)	0.141
BBD ^2^			0.141
No	60 (56.1%)	2 (25.0%)	
Yes	47 (43.9%)	6 (75.0%)	
Laterality			0.453
Unilateral	37 (34.6%)	4 (50.0%)	
Bilateral	70 (94.6%)	4 (50.0%)	
VUR ^3^ Grade			0.028
I	12 (11.2%)	0 (0%)	
II	16 (15.0%)	0 (0%)	
III	48 (44.9%)	3 (37.5%)	
IV	27 (25.2%)	2 (25.0%)	
V	4 (3.7%)	3 (37.5%)	
Console time	146.0 (75–270)	188.5 (171–221)	<0.001
No. of detrusorrhaphy stitches	6 (6–7)	6 (6–6)	0.647
Hospital stay	1 (1–6)	1.5 (1–3)	0.595

^1^ BMI, body mass index (kg/m^2^); ^2^ BBD, bladder bowel dysfunction; ^3^ VUR, vesicoureteral reflux.

**Table 3:** Multivariate logistic regression models in the development cohort.

Variable	Without Intra-and Post-Operative Variable Model				With Intra-and Post-Operative Variable Model			
	β Coefficient	OR	95% CI	*p*-Value	β Coefficient	OR	95% CI	*p*-Value
Age	0.229	1.258	0.995–1.591	0.056	0.43	1.54	1.03–2.29	0.033
BMI ^1^	0.059	1.061	0.865–1.301	0.570	0.02	1.02	0.70–1.51	0.883
BBD ^2^	1.512	4.538	0.699–29.448	0.113	2.35	10.58	0.72–182.15	0.067
VUR ^3^ Grade	1.288	3.627	1.283–10.252	0.015	1.96	7.12	1.19–42.56	0.031
Console time					0.06	1.06	1.01–1.11	0.010
Hospital stay					1.4	4.05	0.10–1.33	0.130

^1^ BMI, body mass index; ^2^ BBD, bladder bowel dysfunction; ^3^ VUR, vesicoureteral reflux.

**Table 4:** VUR resolution prediction by risk group.

Risk Group	Development (*n* = 115) *		Validation (*n* = 46) ^†^	
	*n* (%)	Resolution (%)	*n* (%)	Resolution
Low-risk group (<52 points)	23 (20.0)	100	4 (8.7)	100
Intermediate-risk group (52–70 points)	62 (53.9)	96.8	24 (52.2)	91.7
High-risk group (≥71 points)	30 (26.1)	80	18 (39.1)	77.8

* Fisher’s exact test (*p* = 0.007), **^†^** Fisher’s exact test (*p* = 0.422).

## Data Availability

Not applicable.

## Supplementary Materials

The following are available online at https://www.mdpi.com/article/10.3390/jcm11051327/s1, Table S1: Reclassification table for the model with and without intra- and post-operative variable.
