# Supplementary material for: Development and Validation of a Scoring System for Assessment of Clinical Failure after Pediatric Robot-Assisted Laparoscopic Extravesical Ureteral Reimplantation: A Multi-Center Study
Source: J Clin Med. 2022 Feb 28;11(5):1327. doi: 10.3390/jcm11051327 (PMC8910908; doi:10.3390/jcm11051327)
Supplement: Supplementary file 1 [file jcm-11-01327-s001.zip › jcm-1572305-supplementary.pdf]

Supplementary Table S1. Reclassification table for the model with and without intra- and post-operative variable.

Model without Intra- and Post-Operative Variable Model	Model with Intra- and Post-Operative Variable Model		
	**Low**	**Intermediate**	**High**
Failure (*n* = 8)			
Low	0 (0)	0 (0)	0 (0)
Intermediate	0 (0)	1 (12.5)	1 (12.5)
High	0 (0)	1 (12.5)	5 (62.5)
Success (*n* = 107)			
Low	17 (15.9)	2 (1.9)	0 (0)
Intermediate	6 (5.6)	50 (46.7)	9 (8.4)
High	0 (0)	8 (7.5)	15 (14.0)

Values are numbers (percentages). Event NRI = (0+1)/8-(0+1)/8=12.5%-12.5%=0%. Non-event NRI = (6+50+8)/107-(2+0+9)/107 = (5.6%+46.7%+7.5%)-(1.9%+8.4%)= 49.5%. Category-based NRI = 0.0+0.495 = 0.495 (95% CI,0% and 49.5% of patient who failed and survived, respectively, correctly reclassified by the model with intra- and post-operative variable model.
